# Downregulation of miR-140-3p Contributes to Upregulation of CD38 Protein in Bronchial Smooth Muscle Cells

**DOI:** 10.3390/ijms21217982

**Published:** 2020-10-27

**Authors:** Yoshihiko Chiba, Mayumi Matsumoto, Motohiko Hanazaki, Hiroyasu Sakai

**Affiliations:** 1 Laboratory of Molecular Biology and Physiology, School of Pharmacy, Hoshi University, Tokyo 142-8501, Japan; hoshibiology@gmail.com (M.M.); casensitivity@gmail.com (M.H.); 2 Department of Anesthesiology and Intensive Care Medicine, School of Medicine, International University of Health and Welfare, Chiba 286-8686, Japan; 3 Laboratory of Biomolecular Pharmacology, School of Pharmacy, Hoshi University, Tokyo 142-8501, Japan; sakai@hoshi.ac.jp

**Keywords:** microRNA (miRNA), miR-140-3p, CD38, asthma, bronchial smooth muscle hyperresponsiveness

## Abstract

In allergic bronchial asthma, an increased smooth muscle contractility of the airways is one of the causes of the airway hyperresponsiveness (AHR). Increasing evidence also suggests a possible involvement of microRNAs (miRNAs) in airway diseases, including asthma, although their roles in function and pathology largely unknown. The current study aimed to determine the role of a miRNA, miR-140-3p, in the control of protein expression of CD38, which is believed to regulate the contraction of smooth muscles, including the airways. In bronchial smooth muscles (BSMs) of the mice that were actively sensitized and repeatedly challenged with ovalbumin antigen, an upregulation of CD38 protein concurrently with a significant reduction of miR-140-3p was observed. In cultured human BSM cells (hBSMCs), transfection with a synthetic miR-140-3p inhibitor caused an increase in CD38 protein, indicating that its basal protein expression is regulated by endogenous miR-140-3p. Treatment of the hBSMCs with interleukin-13 (IL-13), an asthma-related cytokine, caused both an upregulation of CD38 protein and a downregulation of miR-140-3p. Transfection of the hBSMCs with miR-140-3p mimic inhibited the CD38 protein upregulation induced by IL-13. On the other hand, neither a CD38 product cyclic ADP-ribose (cADPR) nor its antagonist 8-bromo-cADPR had an effect on the BSM contraction even in the antigen-challenged mice. Taken together, the current findings suggest that the downregulation of miR-140-3p induced by IL-13 might cause an upregulation of CD38 protein in BSM cells of the disease, although functional and pathological roles of the upregulated CD38 are still unclear.

## 1. Introduction

MicroRNAs (miRNAs) are a class of small noncoding single-stranded RNAs that control the expression of complementary target messenger RNAs (mRNAs) [1,2]. The mature miRNAs comprise about 22 nucleotides, derived from long-transcript primary miRNAs (pri-miRNAs) and precursor miRNAs (pre-miRNAs). There is increasing evidence that the mature miRNAs negatively modulate gene expression primarily through base pairing to the 3′ untranslated region (3′-UTR) of target mRNAs, resulting in mRNA cleavage and/or translation repression [1,2,3]. Evidence also suggests a role for miRNAs in a variety of fundamental biological functions, including normal immune function [4] and lung development [5]. In addition, an implication of miRNAs has been suggested in diverse diseases, including airway diseases, such as chronic obstructive pulmonary disease (COPD) [6,7,8,9] and asthma [9,10,11,12,13,14]. It is thus possible that miRNAs are attractive new drug targets.

Increased airway narrowing in response to nonspecific stimuli is a characteristic feature of human obstructive diseases, including bronchial asthma. This abnormality is an important sign of the disease, although the pathophysiological variations leading to the hyperresponsiveness are unclear now. Several mechanisms have been suggested to explain the airway hyperresponsiveness (AHR), such as alterations in the neural control of airway smooth muscle (ASM) [15], increased mucosal secretions [16], and mechanical factors related to remodeling of the airways [17]. In addition, it has also been suggested that one of the factors that contribute to the exaggerated airway narrowing in individuals with asthma is an abnormality of the properties of ASM [18,19,20]. Rapid relief from airway limitation in asthmatic patients by stimulant inhalation may also suggest an involvement of augmented ASM contraction in airway obstruction. It is thus important for the development of asthma therapy to understand changes in the contractile signaling of ASM cells associated with the disease.

Increasing evidence also suggests a possible involvement of miRNAs in the development of AHR [7,10,11,21]. However, their roles in the function and pathology of ASM remain largely unknown. In the present study, we identified multiple miRNAs differentially expressed in bronchial smooth muscles (BSMs) of a murine model of allergic asthma. Among the differentially expressed miRNAs, miR-140-3p was identified as a significantly reduced miRNA. Studies using the miR-140-3p mimic and inhibitor showed CD38 as a target gene in BSM cells. An upregulation of CD38 protein was also demonstrated in BSMs of the murine asthma model used.

## 2. Results

### 2.1. Differentially Expressed miRNAs in BSMs of the Antigen-Induced AHR Mice

To identify miRNAs differentially expressed in BSMs of the antigen-challenged mice, the miRCURY™ LNA miRNA Array (Exiqon A/S) was used. As shown in Table 1, the microarray analyses indicated that 14 mouse miRNAs were differently expressed in BSMs of the antigen-challenged mice as compared to those of control animals. Among them, miR-140-3p, miR-140-5p, and miR-133a-3p were downregulated in the antigen-challenged mice while miR-1971, miR-142-3p, miR-669c-5p, miR-1897-5p, miR-300-5p, miR-1196-5p, miR-302a-3p, miR-133b-3p, miR-1947-3p, miR-3100-3p, and miR-3474 were upregulated (Table 1). Among the differentially expressed miRNAs, the miRBase searches also suggested the expressions of miR-140-3p, miR-140-5p, miR-133a-3p, miR-142-3p, miR-300-5p, miR-302a-3p, and miR-133b-3p in human tissues (Table 1). From the list of differentially expressed miRNAs, we chose miR-140-3p for further investigation because its high expression in human ASM cells and downregulation by inflammatory cytokines have been reported [22,23].

### 2.2. Change in miR-140-3p Level in BSMs of the Antigen-Induced AHR Mice

**Figure 1:**
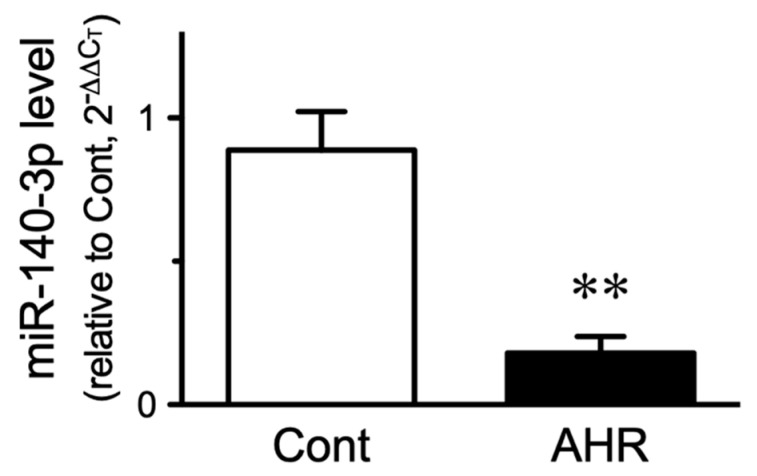
Downregulation of miR-140-3p in bronchial smooth muscles of antigen-induced airway hyperresponsive (AHR) mice. The ovalbumin (OA)-immunized animals were repeatedly challenged with aerosolized OA solution, and total RNAs including miRNAs were extracted from the main bronchi 24 h after the last challenge. The miR-140-3p levels were determined by quantitative real-time reverse transcriptase-polymerase chain reaction. The relative expression of miR-140-3p to U6 snRNA was calculated by the 2^−ΔΔCT^ methods as described in the methods section. Results are presented as mean ± S.E. from six animals, respectively. ** *p* < 0.01 versus control animal (Cont) by unpaired Student’s *t*-test.

To validate the results of the microarray platform, the expression of miR-140-3p was determined by quantitative real-time RT-PCR (RT-qPCR). U6 snRNA was used as an endogenous control. The RT-qPCR analyses successfully detected miR-140-3p and U6 snRNA in BSMs of naive mice: the C_T_ values were 26.11 ± 0.26 and 27.39 ± 0.43, respectively (mean ± S.E., *n* = 6). As shown in Figure 1, miR-140-3p expression was significantly reduced in BSMs of the antigen-challenged mice as compared with those of control animals (*p* < 0.05 by unpaired Student’s *t*-test).

### 2.3. CD38 mRNA as a Predicted Target of miR-140-3p

**Figure 2:**
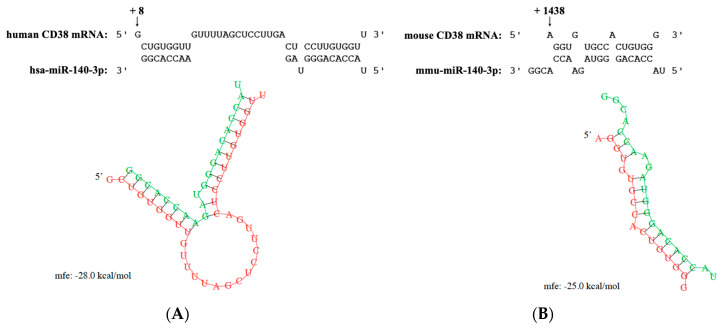
Computational modeling of the interaction between miR-140-3p and the 3′ untranslated region (3′-UTR) of CD38 mRNA in human (**A**) and mouse (**B**) utilizing the RNA hybrid software (http://bibiserv.techfak.uni-bielefeld.de/rnahybrid/). The complementarity between miR-140-3p and 3′-UTR of CD38 mRNA is also shown in each panel. The +8 (**A**) and +1438 (**B**) indicate 8 and 1438 bases downstream from the stop codon of CD38, respectively. *hsa*: *Homo sapiens*, *mmu*: *Mus musculus*, mfe; minimal free energy.

Analysis of targets of miR-140-3p was performed using the public database, microRNA.org (http://www.microRNA.org), which uses an updated version of the miRanda algorithm to find miRNA binding sites in known genes [24]. Since we were interested in studying the role of miRNAs on antigen-induced BSM hyperresponsiveness, we looked for target genes involved with the BSM contraction. Targets having an mirSVR score greater than -0.5 were ignored. As a result, the 3′-UTR of human CD38 mRNA was suggested as a target of *hsa*-miR-140-3p with an mirSVR score of −0.98. The RNA hybrid analysis (http://bibiserv.techfak.uni-bielefeld.de/rnahybrid/) also indicated a strong interaction between miR-140-3p and 3′-UTR of human CD38 mRNA with a minimal free energy (mfe) of −28.0 kcal/mol (Figure 2A). A strong interaction between *mmu*-miR-140-3p (identical with *hsa*-miR-140-3p) and 3′-UTR of mouse CD38 mRNA was also suggested (Figure 2B).

### 2.4. Upregulation of CD38 Protein in BSMs of the Antigen-Induced AHR Mice

**Figure 3:**
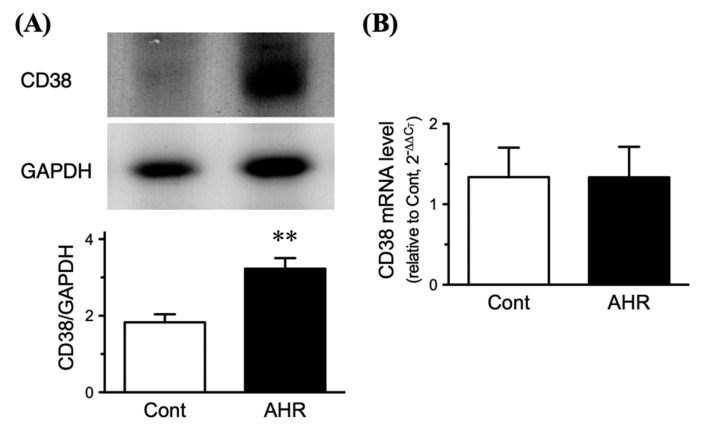
Upregulation of CD38 protein (**A**) but not mRNA (**B**) in bronchial smooth muscles of antigen-induced airway hyperresponsive (AHR) mice. The ovalbumin (OA)-immunized animals were repeatedly challenged with aerosolized OA solution, and proteins and total RNAs including miRNAs were extracted from the main bronchi 24 h after the last challenge. The protein (**A**) and mRNA (**B**) levels of CD38 were determined by immunoblotting and quantitative real-time reverse transcriptase-polymerase chain reaction, respectively. The relative expressions of CD38 to glyceraldehyde 3-phosphate dehydrogenase (GAPDH; (**A**,**B**)) were calculated as described in the methods section. Results are presented as mean ± S.E. from six animals, respectively. ** *p* < 0.01 versus control animal (Cont) by unpaired Student’s *t*-test.

The observations above remind us of an upregulation of CD38 protein in BSMs of the antigen-challenged mice. Immunoblot analyses showed a distinct expression of CD38 in the BSM tissues (Figure 3A, upper panel). In BSMs of the antigen-challenged mice, a significant increase in CD38 protein was observed as compared to those of control animals (Figure 3B, lower panel; *p* < 0.01 by unpaired Student’s *t*-test). On the other hand, real-time RT-qPCR analyses revealed no significant change in the mRNA level of CD38 between the groups (Figure 3B).

### 2.5. Effect of Inhibition of miR-140-3p on CD38 Protein Expression in Cultured Human BSM Cells (hBSMCs)

**Figure 4:**
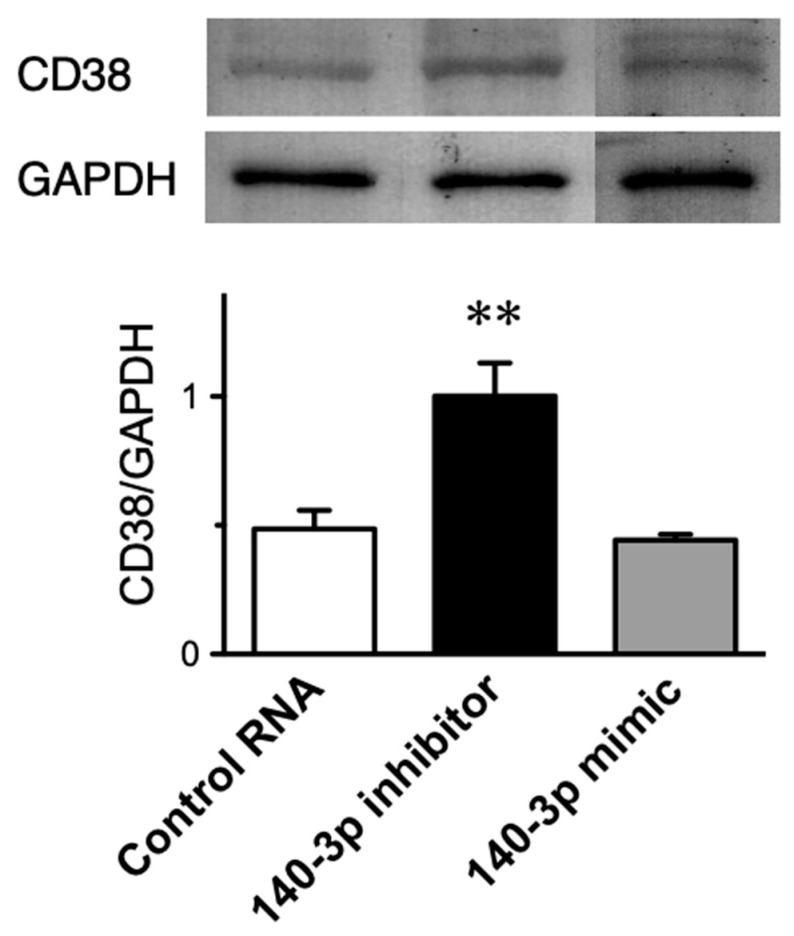
Upregulation of CD38 protein induced by inhibition of miR-140-3p in cultured human bronchial smooth muscle cells. Cells were transfected with a non-targeting 20-25 nt RNA (control RNA), an miR-140-3p inhibitor, or an miR-140-3p mimic as described in the methods section. Total protein samples were prepared 72 h after the transfection and immunoblot analyses were performed. (Upper panel) Representative Western blots. The relative expressions of CD38 to glyceraldehyde 3-phosphate dehydrogenase (GAPDH) were calculated as described in the methods section and the data are summarized in the lower panel. Results are presented as mean ± S.E. from five independent experiments. ** *p* < 0.01 versus the other groups by one-way ANOVA with post hoc Bonferroni/Dunn test.

To determine the role of endogenous miR-140-3p in CD38 expression, hBSMCs were transfected with its synthetic inhibitor, miR-140-3p inhibitor. A non-targeting 20–25 nt RNA was used as a control RNA. As shown in Figure 4, a significant increase in the expression level of CD38 protein (*p* < 0.01 by one-way ANOVA with post hoc Bonferroni/Dunn) was observed in the cells treated with miR-140-3p inhibitor, indicating that basal expression of CD38 protein in the BSM cells is negatively regulated by endogenous miR-140-3p. On the other hand, the miR-140-3p mimic treatment did not affect the CD38 expression (Figure 4). Under these conditions, neither the inhibitor nor mimic of miR-140-3p had an effect on the expression level of CD38 mRNA measured by real-time RT-qPCR (data not shown).

### 2.6. Effect of Inhibition of miR-140-3p on CD38 Protein Exp 2.6. Effects of IL-13 on Expression Levels of CD38 and miR-140-3p in Cultured Human BSM Cells (hBSMCs)

**Figure 5:**
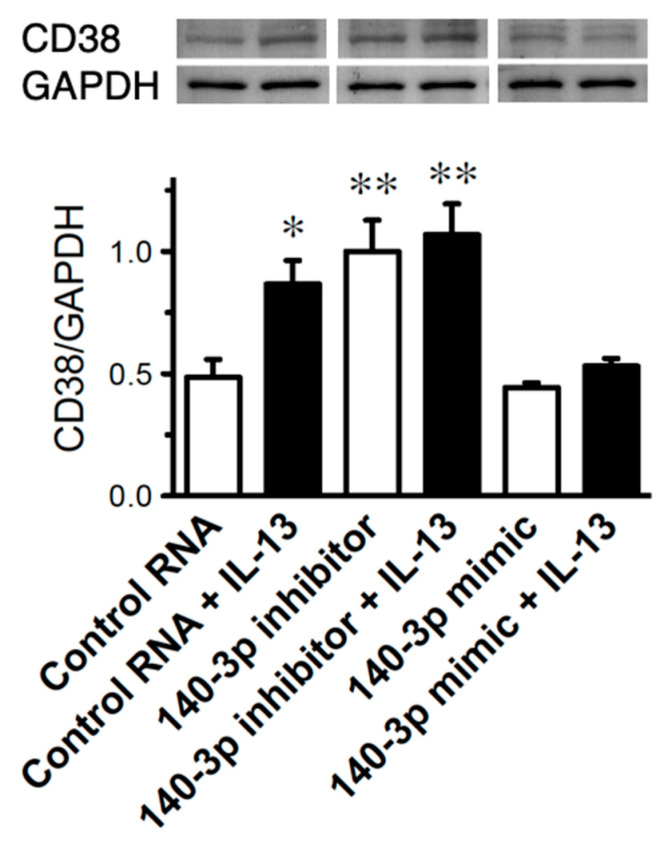
Inhibition of interleukin-13 (IL-13)-induced upregulation of CD38 protein by miR-140-3p mimic in cultured human bronchial smooth muscle cells. Cells were transfected with a non-targeting 20-25 nt RNA (control RNA), an miR-140-3p inhibitor, or an miR-140-3p mimic as described in the methods section, and then treated with IL-13 (100 ng/mL) or its vehicle 48 h after the transfection. Total protein samples were prepared 24 h after the IL-13 treatment and immunoblot analyses were performed. (Upper panel) Representative Western blots. The relative expressions of CD38 to glyceraldehyde 3-phosphate dehydrogenase (GAPDH) were calculated as described in the methods section and the data are summarized in the lower panel. Results are presented as mean ± S.E. from five independent experiments. * *p* < 0.05 and ** *p* < 0.01 versus control RNA only group by one-way ANOVA with post hoc Bonferroni/Dunn test.

**Figure 6:**
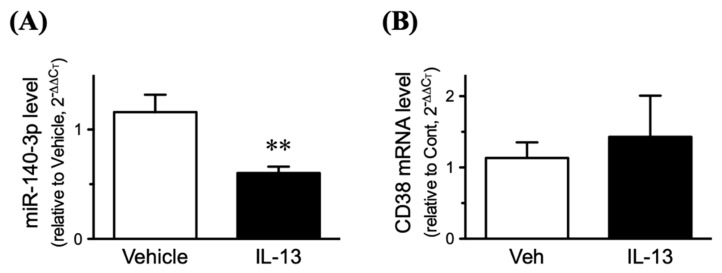
Effects of interleukin-13 (IL-13) on the expressions of miR-140-3p (**A**) and CD38 mRNA (**B**) in cultured human bronchial smooth muscle cells. Cells were treated with IL-13 (100 ng/mL) or its vehicle (Veh) for 24 h and total RNAs including miRNAs were extracted. The gene expressions were determined by quantitative real-time reverse transcriptase-polymerase chain reaction. The relative gene expressions of miR-140-3p to U6 snRNA (**A**) and CD38 to GAPDH mRNAs (**B**) were calculated by the 2^−ΔΔCT^ methods as described in the methods section. Results are presented as mean ± S.E. from six independent experiments. ** *p* < 0.01 versus Veh by unpaired Student’s *t*-test.

Since IL-13 is capable of inducing an upregulation of CD38 in hBSMCs [25], both CD38 and miR-140-3p expressions were determined in the IL-13-treated cells. Consistent with the previous report [25], treatment of the cells with IL-13 caused a significant increase in CD38 protein (Figure 5, “Control RNA” vs. “Control RNA + IL-13” groups: *p* < 0.05 by one-way ANOVA with post hoc Bonferroni/Dunn). Interestingly, the CD38 upregulation induced by IL-13 was abolished in the cells transfected with miR-140-3p mimic, whereas IL-13 caused no further increase in CD38 protein in the cells transfected with miR-140-3p inhibitor (Figure 5). The IL-13 treatment induced a significant decrease in miR-140-3p (Figure 6A: *p* < 0.01 by unpaired Student’s *t*-test) but had no effect on the mRNA expression of CD38 (Figure 6B).

### 2.7. Effects of Cyclic ADP Ribose (cADPR) and Its Antagonist 8-Bromo-cADPR (8-Br-cADPR) on BSM Function

**Figure 7:**
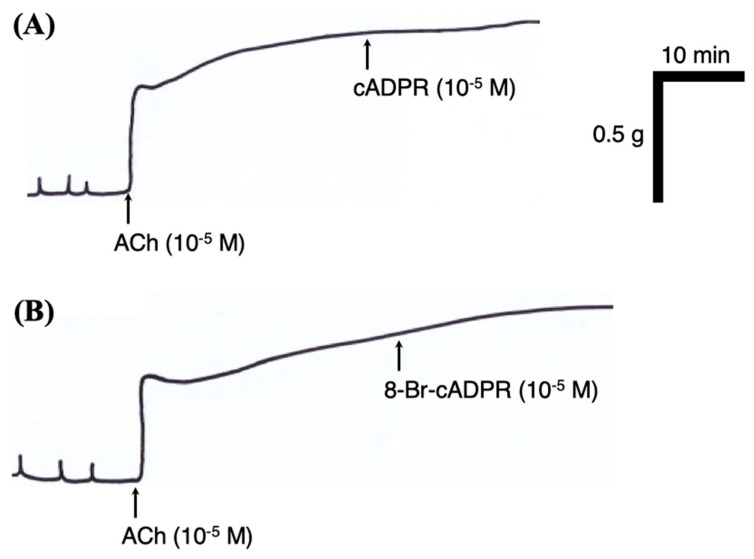
Effects of cyclic ADP-ribose (cADPR, a Ca^2+^ mobilizing mediator generated by CD38; (**A**) and its antagonist 8-bromo-cADPR (8-Br-cADPR; (**B**) on the contraction induced by acetylcholine (ACh) in mouse bronchial smooth muscle tissues of the antigen-challenged mice. After the contraction induced by ACh (10^−5^ M) reached a plateau, cADPR (10^−5^ M; **A**) or 8-Br-cADPR (10^−5^ M; **B**) was applied. The traces are representative of three independent experiments, respectively.

CD38 is known to generate cyclic ADP-ribose (cADPR), which is involved in Ca^2+^ signaling and contraction in ASM [21]. To test the functional role(s) of the upregulated CD38, the effects of cADPR and its antagonist 8-Br-cADPR on BSM contraction were determined using BSM tissues isolated from the mice. Based on the previous reports (e.g., [26]), the 10^−5^ M concentration of each pharmacological agent was used in the present study. Neither exogenously applied cADPR nor 8-Br-cADPR had an effect on the basal resting tone both in the control and antigen-challenged groups (data not shown). In addition, as shown in Figure 7, both the agents had no effect on the contraction induced by acetylcholine (ACh; 10^−5^ M, the concentration that generates about the half-maximal contraction [27]), even in the BSMs isolated from the antigen-challenged group.

## 3. Discussion

In the present study, microarray analysis and RT-qPCR validation revealed a downregulation of miR-140-3p in bronchial smooth muscles (BSMs) of murine experimental asthma (Table 1, Figure 1 and Figure 3). Analyses using public database tools suggested that the 3′ untranslated region (3′-UTR) of CD38 mRNA is one of the targets of miR-140-3p (Figure 2). Inhibition of endogenous miR-140-3p in BSM cells by its synthetic inhibitor caused an upregulation of CD38 (Figure 4), which synthesizes a Ca^2+^-mobilizing messenger cyclic ADP-ribose (cADPR) and has been believed to participate in obstructive pulmonary diseases [21]. Treatment of hBSMCs with IL-13 caused an upregulation of CD38 protein together with a downregulation of miR-140-3p (Figure 5). The IL-13-induced upregulation of CD38 protein was abolished when the cells were transfected with miR-140-3p mimic (Figure 5). These observations indicate that the expression level of CD38 is negatively regulated by endogenous miR-140-3p and that IL-13 could induce CD38 upregulation via an inhibition of miR-140-3p expression. In the repeatedly antigen-challenged mice that possess BSM hyperresponsiveness together with an upregulation of IL-13 in the airways [28,29], a decrease in the level of miR-140-3p concurrently with an upregulation of CD38 in BSM tissue was also found (Figure 1 and Figure 3). Taken together, the downregulation of miR-140-3p induced by IL-13 might cause an upregulation of CD38 in BSM cells of the disease.

Airway smooth muscle (ASM) is an important effector tissue regulating bronchomotor tone. It has been suggested that modulation of ASM by inflammatory mediators, such as cytokines, may play an important role in the development of AHR [30], one of the characteristic features of patients with allergic bronchial asthma. In the BALB/c strain of mice that were actively sensitized and repeatedly challenged using the same procedures as in the current study, an in vivo AHR accompanied by increased IgE production and pulmonary eosinophilia has been demonstrated [31]. In this animal model of allergic bronchial asthma, an increased contractility of isolated BSM to contractile agonists has also been found [32,33]. Although the mechanism of BSM hyperresponsiveness is not fully understood, the current study showed that CD38 protein expression was increased in BSMs of the antigen-challenged mice (Figure 3A).

Our previous findings that the increased BSM contractility observed in the mouse model of allergic asthma were reproduced by IL-13 [28], one of the asthma-related cytokines [34,35], suggest that IL-13 is key for the induction of AHR. So, in the present study, the effect of IL-13 on CD38 expression was determined in the hBSMCs. As a result, treatment of the cells with IL-13 caused a significant increase in CD38 protein (Figure 5, “Control RNA” vs. “Control RNA + IL-13” groups). On the other hand, the IL-13 treatment did not show a significant effect on the mRNA expression of CD38 (Figure 6B), indicating that IL-13 stimulates post-transcriptional regulation of CD38 expression at least in part. Indeed, we showed here that, as also observed in BSMs of antigen-challenged mice, IL-13 caused a downregulation of miR-140-3p (Figure 6A), an miRNA that might interact with the 3′-UTR of CD38 mRNA (Figure 2). In addition, transfection of the cells with miR-140-3p mimic inhibited the CD38 upregulation induced by IL-13 (Figure 5, “Control RNA + IL-13” vs. “140-3p mimic + IL-13” groups). It is thus possible that IL-13 is capable of induction of CD38 protein upregulation by increasing CD38 translation via a downregulation of miR-140-3p in hBSMCs.

To date, miRNAs are considered as one of the fundamental regulatory mechanisms of gene expression. Increasing evidence suggests that dysregulation of miRNA is involved in multiple human diseases, including asthma [36,37]. Among the miRNA families, miR-140-3p has been suggested as a candidate miRNA that contributes to dysfunction of ASM in asthma [23,38,39,40]. Consistent with the report by Kannan and colleagues [38], the current study showed a negative control of the CD38 expression by miR-140-3p in BSM cells. On the other hand, our recent study also demonstrated that a small GTPase RhoA, a Ca^2+^-sensitizing protein that contributes to the augmented BSM contraction in allergic asthma [41], is one of the targets of miR-140-3p [14]. It is thus possible that miR-140-3p controls the expression of multiple proteins in ASM to regulate its contractility. In support, it has been suggested that one miRNA can target multiple genes within one cell type [40,42].

We postulated that inhibition of endogenous miR-140-3p could further augment the upregulation of CD38 protein induced by IL-13. To test the hypothesis, the hBSMCs treated with miR-140-3p inhibitor were incubated with IL-13. However, IL-13 did not show a significant increase in the level of CD38 protein in the cells transfected with miR-140-3p inhibitor, although a tendency to increase was observed (Figure 5, “140-3p inhibitor” vs. “140-3p inhibitor + IL-13” groups). The results might be consistent with the previous report that no further IL-13-induced upregulation of RhoA was found in the cells treated with an inhibitor of miR-133a-3p, an miRNA that negatively regulates the RhoA protein expression [10]. Probably, it might be due to a ceiling effect on the protein expression in the cells. On the other hand, transfection of the hBSMCs with miR-140-3p mimic did not exhibit an inhibitory effect on CD38 expression (Figure 4, “Control RNA” vs. “140-3p mimic” groups), indicating that the basal CD38 level is controlled by endogenous miR-140-3p effectively in the cells.

Although the mechanism of reduction of miR-140-3p induced by antigen challenge (Figure 1) or IL-13 (Figure 6A) is unclear now, the finding that the expression level of miR-140-5p was also decreased (Table 1) suggests an idea that the transcription of the *mir-140* gene might be reduced by the stimulation. In general, the miRNA gene is transcribed into a primary miRNA (pri-miRNA), and then processed into precursor miRNA (pre-miRNA). The pre-miRNA is then processed into a short RNA duplex termed miRNA duplex, and mature single-stranded miRNA, such as miR-140-3p and miR-140-5p, is finally generated. On the other hand, it has also been demonstrated that long non-coding RNAs (lncRNAs) can act as miRNA sponges to reduce the miRNA regulatory effect on its target genes. In this regard, lncRNAs, such as TRPM2-AS [43], SNHG1 [44], and ANCR [45], are reported to inhibit miR-140-3p. Further studies, including the effects of cytokines other than IL-13, are needed to make clear the mechanism of downregulation of miR-140-3p.

CD38 is an ectoenzyme that catalyzes the synthesis of cADPR, a metabolite of nicotinamide adenine dinucleotide (NAD) with Ca^2+^-mobilizing activity [46]. cADPR is degraded to ADP ribose (ADPR), the biologically inactive compound, by the enzyme cADPR hydrolase [47]. In smooth muscles of the airways, the application of cADPR stimulated Ca^2+^ release [47,48] and potentiated the contraction induced by ACh [48]. In addition, the ASM cells of CD38-deficient mice exhibited an attenuated Ca^2+^ release induced by agonists [49]. A reduced airway responsiveness to inhaled methacholine was also demonstrated in the CD38-deficient mice [50]. Furthermore, the CD38-deficient mice failed to develop the AHR induced by allergen, IL-13, or TNFa challenge [50,51,52]. It is thus possible that the increased CD38 expression (Figure 3 A) is a cause of the BSM hypercontraction observed in this animal model of asthma [27,28,32]. However, contrary to our expectation, neither basal tone nor the ACh-induced contraction were affected by cADPR and its antagonist 8-Br-cADPR even in the BSMs of antigen-challenged mice (Figure 7). The results might be consistent with previous reports that cADPR had no effect on Ca^2+^ release in guinea pig vas deferens and aorta [53] and contraction in rabbit tracheal smooth muscle [54]. In a rat model of allergic asthma that has BSM hypercontraction, no significant change in the agonist-induced increase in Ca^2+^ concentration was also demonstrated [55]. Further studies are needed to clarify the functional role of upregulated CD38 in allergic airway diseases.

In conclusion, the current study demonstrated that the CD38 expression is negatively regulated by miR-140-3p in BSM cells and that miR-140-3p is downregulated concurrently with an upregulation of CD38 in BSMs of experimental asthma. IL-13, an asthma-related cytokine, is capable of reducing miR-140-3p to cause an increase in CD38 expression. These findings strongly support previous reports by Kannan and colleagues [38,56], although the exact roles of CD38 in ASM contraction and hyperresponsiveness still remain unclear.

## 4. Materials and Methods

### 4.1. Mouse Model of Allergic Bronchial Asthma

Male BALB/c mice were purchased from the Charles River Japan, Inc. (Kanagawa, Japan) and housed in a pathogen-free facility. All animal experiments were approved by the Animal Care Committee of the Hoshi University (Tokyo, Japan).

Preparation of a murine model of allergic bronchial asthma, which has an in vivo AHR [31], was performed as described previously [32]. In brief, BALBc mice (8 weeks of age) were actively sensitized by intraperitoneal injections of 8 µg ovalbumin (OA; Seikagaku Co., Tokyo, Japan) with 2 mg Imject Alum (Pierce Biotechnology, Inc., Rockfold, IL, USA) on days 0 and 5. The sensitized mice were challenged with aerosolized OA-saline solution (5 mg/mL) for 30 min on days 12, 16, and 20. A control group of mice received the same immunization procedure, and inhaled saline aerosol instead of OA challenge. The aerosol was generated with an ultrasonic nebulizer (Nihon Kohden, Tokyo, Japan) and introduced to a Plexiglas chamber box (130 × 200 mm, 100 mm height) in which the mice were placed. Twenty-four hours after the last OA challenge, mice were sacrificed by exsanguination from the abdominal aorta under urethane (1.6 g/kg, *i.p*.; Sigma & Aldrich, St. Louis, MO, USA) anesthesia. About a 3-mm length of the left main bronchial ring without epithelium was isolated [28], immediately disrupted with 1x sodium dodecyl sulfate (SDS) sample buffer (100 µL/tissue), and used for Western blot analyses. Total RNAs containing miRNAs were extracted using a Vantage^TM^ total RNA purification kit (OriGene, Rockville, MD, USA) according to the manufacturer’s instructions. In another set of experiments, the bronchial rings were used for the contraction study as described below.

### 4.2. miRNA Microarray Analyses

miRNA microarray services were contracted out to Cosmo Bio Co., Ltd. (Tokyo, Japan). RNA quality was assessed using the Agilent Bioanalyzer 2100 (Agilent Technologies, Inc., Santa Clara, CA, USA). Total RNAs (130 ng) from samples and references were labeled with Hy3 and Hy5 fluorescent label, respectively, using the miRCURY™ LNA Array power labeling kit (Exiqon A/S, Vedbaek, Denmark) following the procedure described by the manufacturer. The Hy3-labeled samples and a Hy5-labeled reference RNA sample were mixed pair-wise and hybridized to the miRCURY™ LNA miRNA Array (Exiqon A/S). The hybridization was performed according to the miRCURY™ LNA array manual. After hybridization, the miRCURY™ LNA array microarray slides were scanned using the Agilent G2505C Microarray Scanner System (Agilent Technologies, Inc.) using the Agilent Scan Control software (Agilent Technologies, Inc.). The microarray images were analyzed with Feature Extraction Software (Ver.10.7.3.1, Agilent Technologies, Inc.). The standard of statistical significance was the corrected ratios of hybridization signal intensity between the OA-challenged mice and control animals. miRNAs were considered differently expressed if their ratios were more than 2.0 or less than 0.5.

### 4.3. Cell Culture and Sample Collection

Normal human bronchial smooth muscle cells (hBSMCs; Cambrex Bio Science Walkersville, Inc., Walkersville, MD, USA) were maintained in SmBM medium (Cambrex) supplemented with 5% fetal bovine serum, 0.5 ng/mL human epidermal growth factor (hEGF), 5 µg/mL insulin, 2 ng/mL human fibroblast growth factor-basic (hFGF-b), 50 µg/mL gentamicin, and 50 ng/mL amphotericin B. Cells were maintained at 37 °C in a humidified atmosphere (5% CO_2_), fed every 48-72 h, and passaged when cells reached 90–95% confluence. Then, the hBSMCs (passages five through seven) were seeded in 6-well plates (Becton Dickinson Labware, Franklin Lakes, NJ, USA) at a density of 3500 cells/cm^2^ and, when 80–85% confluence was observed, cells were cultured without serum for 24 h before addition of recombinant human IL-13 (100 ng/mL; PeproTech EC, Ltd., London, UK). At the indicated time after the IL-13 treatment, cells were washed with phosphate-buffered saline, immediately collected and disrupted with 1x SDS sample buffer (150 µL/well), and used for Western blot analyses. Total RNAs containing miRNAs were extracted using a Vantage^TM^ total RNA purification kit (OriGene) according to the manufacturer’s instructions.

### 4.4. Transfection of miR-140-3p Inhibitor and Mimic

The hBSMCs were plated at a density of 2 × 10^5^ cells/well in a 6-well plate with SmGM medium. The next day, cells were transfected with 100 pmol (final 40 nM) of either an inhibitor of miR-140-3p (Peptide Nucleic Acids (PNAs^TM^) miRNA inhibitor; Cat. No.: PI-1171; Panagene Inc., Daejeon, Korea), a mimic of miR-140-3p (miCENTURY OX miNatural; Cat. No.: HN0000140A1-2; Cosmo Bio Co., Ltd.), or a control RNA (Cosmo Bio Co., Ltd.) using the Lipofectamine^TM^ 2000 transfection reagent (Invitrogen, Carlsbad, CA, USA) according to the manufacturer’s instructions. After a 4-h transfection in Opti-MEM^TM^ I reduced serum medium (Invitrogen), cells were cultured in SmGM medium for 20 h and then in serum-free SmGM medium. Forty-eight hours after the transfection, IL-13 (100 ng/mL) or its vehicle (water) was administered to the cells.

### 4.5. Quantitative RT-PCR Analyses

To synthesize cDNAs, 200 ng of total RNAs were poly-adenylated and cDNAs were synthesized using a miRCURY LNA^TM^ Universal cDNA Synthesis Kit (Exiqon A/S) according to the manufacturer’s instructions. Then, the RT reaction mixture (1 µL) was subjected to real-time PCR analyses using a StepOnePlus^TM^ real-time PCR system (Applied Biosystems, Foster City, CA, USA) with Fast SYBR Green Master Mix (Applied Biosystems) according to the manufacturer’s instructions. The reactions were incubated in a 96-well optical plate at 95 °C for 20 s, following by 43 cycles of 95 °C for 3 s and 60 °C for 30 s. The primer sets used were: LNA^TM^ PCR primer sets for mouse and human miR-140-3p and U6 snRNA (Exiqon A/S), 5′-AAAGGACTGCAGCAACAACC-3′ (sense) and 5′-CCATTGAGCATCACATGGAC-3′ (antisense) for human CD38, 5′-CCCACTCCTCCACCTTTGAC-3′ (sense) and 5′-CCCTGTTGCTGTAGCCAAATTC-3′ (antisense) for human GAPDH, 5′-GCCCACATTGGAGTGAAAAC-3′ (sense) and 5′-TTGAGCATCACTTGGACCAC-3′ (antisense) for mouse CD38, and 5′-CCTCGTCCCGTAGACAAAATG-3′ (sense), and 5′-TCTCCACTTTGCCACTGCAA-3′ (antisense) for mouse GAPDH.

### 4.6. Western Blot Analyses

Protein samples were subjected to 15% sodium dodecyl sulfate-polyacrylamide gel electrophoresis and the proteins were then electrophoretically transferred to a PVDF membrane (WSE-4051CP; Atto, Co., Tokyo, Japan). After blocking with EzBlock Chemi^TM^ (Atto, Co.), the PVDF membrane was incubated with polyclonal rabbit anti-CD38 (1:1000 dilution; Santa Cruz Biotechnology, Inc., Santa Cruz, CA, USA) antibody. Then, the membrane was incubated with horseradish peroxidase-conjugated donkey anti-rabbit IgG (1:2500 dilution; Santa Cruz Biotechnology, Inc.), detected by EzWestBlue^TM^ (Atto, Co.), and analyzed by a densitometry system. Detection of the house-keeping gene was also performed on the same membrane by using monoclonal mouse anti-GAPDH (1:10,000 dilution; Santa Cruz Biotechnology, Inc.) to confirm the same amount of proteins loaded.

### 4.7. Data and Statistical Analyses

In the real-time PCR analyses, the comparative threshold cycle (C_T_) method was used for relative quantification of the target genes. Differences in the C_T_ values (ΔC_T_) between miR-140-3p and U6 snRNA or CD38 and GAPDH were calculated to determine the relative expression levels, using the following formula: ΔΔC_T_ = (ΔC_T_ of the treated sample) − (ΔC_T_ of the control sample). The relative expression level between the samples was calculated according to the equation 2^−ΔΔCT^.

All the data are expressed as the mean ± S.E. Statistical significance of difference was determined by unpaired Student’s *t*-test or one-way analysis of variance (ANOVA) with post hoc Bonferroni/Dunn (Prism^TM^ 5 for Mac OS X; GraphPad Software, Inc., La Jolla, CA, USA). A value of *p* < 0.05 was considered significant.

## Figures and Tables

**Table 1:** List of differentially expressed microRNAs (miRNAs) in bronchial smooth muscle of antigen-induced airway hyperresponsive (AHR) mice.

miRNA (*mmu*)	Accession	Mature Sequence	Human Expression	AHR/Cont
miR-140-3p	MIMAT0000152	5′-UACCACAGGGUAGAACCACGG-3′	Yes	0.448
miR-140-5p	MIMAT0000151	5′-CAGUGGUUUUACCCUAUGGUAG-3′	Yes	0.449
miR-133a-3p	MIMAT0000145	5′-UUUGGUCCCCUUCAACCAGCUG-3′	Yes	0.465
miR-1971	MIMAT0009446	5′-GUAAAGGCUGGGCUGAGA-3′	No	2.004
miR-142-3p	MIMAT0000155	5′-UGUAGUGUUUCCUACUUUAUGGA-3′	Yes	2.101
miR-669c-5p	MIMAT0003479	5′-AUAGUUGUGUGUGGAUGUGUGU-3′	No	2.123
miR-1897-5p	MIMAT0007864	5′-CUUUGGAUGGAGAAAGAGGGGG-3′	No	2.257
miR-300-5p	MIMAT0004578	5′-UUGAAGAGAGGUUAUCCUUUGU-3′	Yes	2.262
miR-1196-5p	MIMAT0005857	5′-AAAUCUACCUGCCUCUGCCU-3′	No	2.488
miR-302a-3p	MIMAT0000380	5′-UAAGUGCUUCCAUGUUUUGGUGA-3′	Yes	2.525
miR-133b-3p	MIMAT0000769	5′-UUUGGUCCCCUUCAACCAGCUA-3′	Yes	3.134
miR-1947-3p	MIMAT0017343	5′-GCACUGAGCUAGCUCUCCCUCC-3′	No	6.173
miR-3100-3p	MIMAT0014920	5′-CUGUGACACACCCGCUCCCAG-3′	No	6.410
miR-3474	MIMAT0015646	5′-CCCUGGGAGGAGACGUGGAUUC-3′	No	12.195

The accession numbers, mature sequences and human expression are from the miRBase (http://microrna.sanger.ac.uk/). *mmu*: *Mus musculus*.

## Abbreviations

ACh	acetylcholine
AHR	airway hyperresponsiveness
ANOVA	analysis of variance
ASM	airway smooth muscle
BSM	bronchial smooth muscle
cADPR	cyclic ADP-ribose
COPD	chronic obstructive pulmonary disease
C_T_	threshold cycle
GAPDH	glyceraldehyde-3-phosphate dehydrogenase
hBSMC	human bronchial smooth muscle cell
hEGF	human epidermal growth factor
hFGF-b	human fibroblast growth factor-basic
IL-13	interleukin-13
LNA	locked nucleic acid
miRNA	microRNA
mRNA	messenger RNA
NAD	nicotinamide adenine dinucleotide
OA	ovalbumin
pre-miRNA	precursor miRNA
PCR	polymerase chain reaction
pri-miRNA	primary miRNA
PVDF	polyvinylidene difluoride
RT	reverse transcription
RT-qPCR	quantitative real-time RT-PCR
snRNA	small nuclear RNA
UTR	untranslated region
